# AT1-receptor-deficiency induced atheroprotection in diabetic mice is partially mediated via PPARγ

**DOI:** 10.1186/1475-2840-12-30

**Published:** 2013-02-01

**Authors:** Vedat Tiyerili, Ulrich M Becher, Adem Aksoy, Dieter Lütjohann, Sven Wassmann, Georg Nickenig, Cornelius FH Mueller

**Affiliations:** 1 Medizinische Klinik und Poliklinik II, Innere Medizin, Universitätsklinikum Bonn, Sigmund Freud Str. 25, 53105, Bonn, Germany; 2 Institut für Klinische Chemie und Pharmakologie, Universitätsklinikum Bonn, Sigmund Freud Str. 25, 53105, Bonn, Germany; 3 Isar Herzzentrum München, Sonnenstrasse 24-26, 80331, Munich, Germany

**Keywords:** Diabetes mellitus, Atherosclerosis, Angiotensin, Receptors

## Abstract

**Objective:**

Peroxisome-proliferator–activated-receptor-γ (PPARγ) acts as a transcriptional regulator of multiple genes involved in glucose and lipid metabolism. In vitro studies showed that activated PPARγ suppresses AT1R-gene expression and vice versa. However, it has not yet been determined in vivo, whether AT1R-PPARγ-interactions play a relevant role in the pathogenesis of diabetic complications and specifically in accelerated atherosclerosis.

**Methods and results:**

ApoE^−/−^ and ApoE^−/−^/AT1R^−/−^-mice were rendered diabetic by intraperitoneal injections of streptozotocin. Diabetic and non-diabetic ApoE^−/−^-mice were further randomized to receive the AT1R antagonist telmisartan, the selective PPARγ antagonist GW9662, telmisartan and GW9662 or vehicle for 18 weeks. Diabetic and non-diabetic ApoE^−/−^/AT1R^−/−^-mice were randomized to receive either GW9662 or vehicle. GW9662 treatment in diabetic ApoE^−/−^ and diabetic ApoE^−/−^/AT1^−/−^-mice resulted in the highest elevation of fasting blood glucose levels, whereas telmisartan treatment and AT1 deficiency in ApoE^−/−^-mice showed the lowest fasting blood glucose levels. Diabetic ApoE^−/−^-mice displayed severe impairment of endothelial function, enhanced oxidative stress and increased atherosclerotic lesion formation. ApoE^−/−^/AT1R^−/−^ and telmisartan-treated ApoE^−/−^-mice showed a significantly better endothelial function, decreased oxidative stress and reduced atherosclerotic lesion formation. Treatment of diabetic ApoE^−/−^ and ApoE^−/−^/AT1R^−/−^-mice with the selective PPARγ antagonist GW9662 omitted the atheroprotective effects of AT1R deficiency or AT1 antagonism.

**Conclusion:**

Genetic disruption or pharmacological inhibition of the AT1R attenuates atherosclerosis and improves endothelial function in diabetic ApoE^−/−^-mice via the PPARγ pathway.

## Introduction

Diabetes mellitus is a leading cause of morbidity and mortality in western countries due to cardiovascular complications [1]. It has been suggested that hyperglycaemia, insulin resistance, glycation of proteins, oxidative stress and inflammation may be related to atherogenesis in diabetes [2]. The metabolic abnormalities associated with diabetes lead to activation of the renin-angiotensin-aldosterone system (RAAS) with a subsequent increase of angiotensin II (Ang II) and increased AT1-receptor (AT1R) activation [3,4]. Increased AT1R activation promotes formation of reactive oxygen species (ROS) which are in turn closely linked to the onset and progression of endothelial dysfunction and atherogenesis [5]. Inhibitors of the RAAS system are associated with improvement of insulin sensitivity, reduced rates of new onset of diabetes and decreased ROS formation [6-8]. So far, the causal link between these clinical observations and AT1R inhibition remains unclear. Some angiotensin receptor blockers (ARBs), such as telmisartan are partial agonists of peroxisome proliferator-activated receptors (PPARs) [9-11]. The most abundant isoform, PPARγ, plays an important role in the regulation of adipogenesis and insulin sensitivity [12]. Furthermore, PPARγ activation has been associated with anti-atherosclerotic effects including reduced formation of ROS [13]. Beneficial effects of ARBs may be partially attributed to the activation of PPARγ [9]. *In vitro* studies investigating the interaction of PPARγ and the AT1R in vascular smooth muscle cells (VSMC) showed that activated PPARγ suppresses AT1R gene expression and vice versa, suggesting that pharmacological blockade or genetic disruption of the AT1R leads to enhanced PPARγ activity thereby mediating anti-atherosclerotic effects in the vascular compartment [14,15]. However, the relevance of these mechanisms has not been determined in an *in vivo* model of diabetes. Whether interactions of AT1R and PPARγ play a key role in the pathogenesis of diabetes-induced atherosclerosis remains undetermined.

In the present study we analysed the influence of AT1R-PPARγ interactions on diabetic-induced atherosclerotic lesion formation and endothelial function in an experimental long-term diabetic mouse model. In this well characterized model, injection of the cytotoxin streptozotocin (STZ) results in a reduction in ß-cells and an increase in plasma glucose to diabetic levels [4]. The validity of this model has recently been confirmed as appropriate for the study of diabetes-associated atherosclerosis by the National Institutes of Health (NIH)/Juvenile Diabetes Research Foundation (JDRF)-supported Animal Models of Diabetic Complications Consortium [16]. Our aim was to determine whether pharmacological inhibition or genetic disruption of the AT1R and the PPARγ pathway would interfere with the pathogenesis of diabetic vascular complications.

## Methods

### Animals and treatment protocols

**Figure 1:**
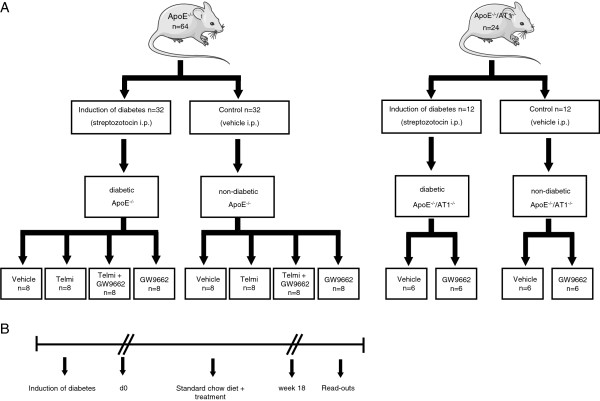
**Experimental setting.** (**A**) Thirty-two ApoE^−/−^-mice and 12 ApoE^−/−^/AT1R^−/−^-mice were rendered diabetic after injections of streptozotocin. The same quantity of ApoE^−/−^ and ApoE^−/−^/AT1R^−/−^-mice received vehicle alone and served as non-diabetic controls. Diabetic and non-diabetic ApoE^−/−^-mice were further randomized in groups of 8 animals to receive telmisartan, GW9662, telmisartan and GW9962 or vehicle for 18 weeks. Diabetic and non-diabetic ApoE^−/−^/AT1R^−/−^-mice were further randomized in groups of 6 animals to receive either GW9662 or vehicle for 18 weeks. (**B**) After treatment of 18 weeks mice were sacrificed and read-outs were performed.

Female, 6-week-old homozygous apolipoprotein E deficient (ApoE^−/−^) mice (genetic background: C57BL/6J, Charles River, Sulzfeld, Germany) and AT1A receptor knockout mice (AT1R^−/−^) with identical genetic background (kindly provided by Dr. Coffmann, University of North Carolina) were used for this study. Thirty-two ApoE^−/−^-mice and 12 ApoE^−/−^/AT1R^−/−^-mice were rendered diabetic by 5 daily intraperitoneal injections of streptozotocin (Sigma-Aldrich, Germany) at a dose of 55mg/kg in citrate buffer or received citrate buffer (0.01 mol/l, pH: 4.5) alone (Figure 1A). All streptozotocin treated animals had blood glucose-levels ≥250 mg/dl 14 days after the induction of diabetes. The same number of ApoE^−/−^-mice and ApoE^−/−^/AT1R^−/−^ served as non-diabetic control animals (Figure 1A). In addition, diabetic and non-diabetic ApoE^−/−^-mice were randomized in 8 groups consisting of 8 animals to receive the AT1R-blocker telmisartan (Sigma-Aldrich, Germany) at a dose of 40 mg/kg body weight per day orally via chow or the selective PPARγ antagonist GW9662 (Sigma-Aldrich) i.p. at a dose of 1mg/kg body weight every second day or telmisartan and GW9962 or vehicle for 18 weeks (Figure 1A). Diabetic and non-diabetic ApoE^−/−^/AT1R^−/−^-mice were further randomized in 4 groups consisting of 6 animals to receive either GW9662 or vehicle for 18 weeks (Figure 1A). After induction of diabetes the animals were treated for 18 weeks, had unrestricted access to water and standard mouse chow and were maintained in a room with a 12-hour light/dark cycle and a constant temperature of 22°C. The experimental setting is depicted as flow chart in Figure 1B. After treatment of 18 weeks mice were sacrificed and read-outs were performed (Figure 1B). All animal experiments were performed in accordance with institutional guidelines and the German animal protection law.

### Measurements of blood pressure (BP), heart rate, blood glucose and body weight

Systolic blood pressure and heart rate were measured by a computerized tail-cuff system (CODA 6, Kent Scientific) in conscious animals. Blood glucose levels were measured using Accu-Chek®-Sensor, Roche, Mannheim, Germany). Blood samples were collected by tail vein puncture. Body weights were measured weekly and changes in body weight at baseline compared to body weight after 18 weeks of treatment were calculated.

### Aortic ring preparations and tension recording

After excision of the descending aorta, the vessel was immersed in chilled, modified Tyrode buffer containing, in mmol/L, NaCl 118.0, CaCl2 2.5, KCl 4.73, MgCl2 1.2, KH2PO4 1.2, NaHCO3 25.0, Na EDTA 0.026, D(+) glucose 5.5, pH 7.4. Three-millimeter rings were mounted in organ baths filled with the above-described buffer (37°C; continuously aerated with 95% O2 and 5% CO2) and were attached to a force transducer, and isometric tension was recorded. The vessel segments were gradually stretched over 60 minutes to a resting tension of 10 mN. The drug concentration was increased when vasoconstriction or vasorelaxation was completed. Drugs were washed out before the next substance was added.

### Staining of atherosclerotic lesions and morphometric analysis

Hearts with ascending aortas were embedded in Tissue Tek OCT embedding medium and sectioned on a Leica cryostat (9 μm), starting at the apex and progressing through the aortic valve area into the ascending aorta and the aortic arch and placed on poly-L-lysine (Sigma) coated slides. At least 15 consecutive sections per animal were used for analysis. For detection of atherosclerotic lesions, aortic cryosections were fixed with 3.7% formaldehyde and stained with oil red O working solution. For morphometric analysis, hematoxylin staining was performed according to standard protocols. Stained samples were examined with a Zeiss Axiovert 200 microscope (Carl Zeiss Jena, Germany) and an AxioCam MRc5. Images were acquired with Zeiss AxioVision software Rel. 4.5.0 and processed with Corel Graphic Suite X4. For quantification of atherosclerotic plaque formation in the aortic root, lipid staining area and total area of serial histological sections were measured. Atherosclerosis data are expressed as lipid-staining area in percent of total surface area. The investigator who performed the histological analyses was unaware of the hypothesis of this study and the treatment of the respective animal group.

### Measurement of vascular reactive oxygen species

ROS release in intact aortic segments was determined by L-012 chemiluminescence, as previously described [5]. Chemiluminescence was assessed over 15 minutes in a scintillation counter (Lumat LB 9501, Berthold) at 1-minute intervals. The vessel segments were then dried, and dry weight was determined. ROS release is expressed as relative chemiluminescence per milligram of aortic tissue.

### Statistical analysis

Data are presented as mean ± SEM. Statistical analysis was performed using the ANOVA test followed by the Neuman–Keuls post hoc analysis. *P* < 0.05 indicates statistical significance.

## Results

### Blood pressure, heart rate and metabolic parameters

Total cholesterol levels, fasting blood glucose, change in body weight, blood pressure and heart rate were measured in all groups (Table 1). Mice were treated as described in the method section and depicted in Figure 1A and Figure 1B. Blood pressure (mmHg) and heart rates (bpm) were measured in all groups by tail-cuff measurements. After 18 weeks systolic blood pressure was reduced in ApoE^−/−^/AT1^−/−^-mice compared to ApoE^−/−^-mice. Heart rates were significant higher in diabetic animals. Measurements of fasting blood glucose levels showed pathological glucose levels in diabetic animals compared to non-diabetic animals. GW9662 treatment in diabetic ApoE^−/−^ and diabetic ApoE^−/−^/AT1^−/−^-mice resulted in the highest elevation of fasting blood glucose levels. Cotreatment with telmisartan and GW9662 markedly attenuated this effect in diabetic ApoE^−/−^-mice. Diabetic ApoE^−/−^-mice treated with telmisartan and diabetic ApoE^−/−^/AT1^−/−^-mice showed the lowest fasting blood glucose levels compared to the other diabetic animals. At baseline (d0) body weight was identical in all groups (data not shown). After 18 weeks of standard chow diet, all groups of diabetic ApoE^−/−^-mice had significant more loss of body weight than the corresponding groups of diabetic ApoE^−/−^/AT1R^−/−^-mice. Administration of GW9662 led to a significant decrease in body weight in diabetic ApoE^−/−^ and diabetic ApoE^−/−^/AT1R^−/−^-mice compared to vehicle treated diabetic ApoE^−/−^ and diabetic ApoE^−/−^/AT1R^−/−^-mice. Interestingly, GW9662 treated diabetic ApoE^−/−^-mice lost significant more body weight then GW9662 treated diabetic ApoE^−/−^/AT1R^−/−^-mice. In contrast, all groups of non-diabetic ApoE^−/−^-mice and non-diabetic ApoE^−/−^/AT1R^−/−^-mice had a uniform increment in body weight after 18 weeks. Total cholesterol levels were higher in diabetic ApoE^−/−^-mice and diabetic ApoE^−/−^/AT1^−/−^-mice compared to non-diabetic groups. Highest total cholesterol levels were detected in GW9662 treated diabetic ApoE^−/−^-mice, indicating poor glucose metabolism and increased lipolysis in GW9662 treated diabetic ApoE^−/−^-mice. Co-treatment with telmisartan reduced this effect significantly. GW9662 had no effect in diabetic ApoE^−/−^/AT1^−/−^-mice. All parameters are shown in Table 1.

**Table 1:** **Blood pressure**, **heart rate and metabolic parameters**

	**Total cholesterol (mmol/l)**	**Fasting blood glucose (mmol/l)**	**Change in body weight (%)**	**Systolic BP (mmHg)**	**Heart rate (beats/min)**
**Diabetic ApoE**^−/−^					
Vehicle (n = 8)	29 ± 3^*,#^	23 ± 1^*,#^	−5,2 ± 0.5^*,#^	124 ± 4^#^	992 ± 32^*^
Telmisartan (n = 8)	31 ± 5^*^	19 ± 1^*^	−6,9 ± 0.3^*^	132 ± 9	710 ± 91
Telmi + GW9662 (n = 7)	48 ± 7^*‡^	22 ± 0.5^*^	−3.4 ± 0.6^*^	124 ± 5	887 ± 66^*^
GW9662 (n = 7)	72 ± 12^*,#,‡^	32 ± 1^*,#,‡^	−12.4 ± 0.6^*,#,‡^	123 ± 7^#^	1040 ± 55^*,#^
**Diabetic ApoE**/**AT1**^−/−^					
Vehicle (n = 6)	15 ± 5^*^	12 ± 1^*^	12.6 ± 0.7^*^	106 ± 2	884 ± 70
GW9662 (n = 6)	17 ± 3^*^	19 ± 2^*,#^	−5.9 ± 0.9^*,#^	106 ± 4	772 ± 38
**Non**-**diabetic ApoE**^−/−^					
Vehicle (n = 8)	20 ± 2	5 ± 0.2	46 ± 0.8	123 ± 3	649 ± 38
Telmisartan (n = 8)	21 ± 3	5 ± 0.4	40 ± 0.3	118 ± 4	727 ± 21
Telmi + GW9662 (n = 8)	22 ± 2	5 ± 0.3	42 ± 0.5	125 ± 4	708 ± 42
GW9662 (n = 8)	18 ± 1	4 ± 0.4	48 ± 0.3	131 ± 3	695 ± 13
**Non**-**diabetic ApoE**/**AT1**^−/−^					
Vehicle (n = 6)	8 ± 3	5 ± 1	47 ± 0.6	102 ± 5^*^	742 ± 46
GW9662 (n = 6)	8 ± 3	4 ± 1	50 ± 0.5	105 ± 5^*^	679 ± 50

To determine cardio-metabolic effects systolic BP, heart rate, fasting blood glucose levels, change in body weight and total cholesterol levels were assessed in vehicle treated diabetic and non-diabetic ApoE^−/−^ and ApoE^−/−^/AT1R^−/−^-mice. ^*^P < 0.05 vs. non-diabetic groups. ^#^p < 0.05 vs. corresponding diabetic ApoE^−/−^/AT1R^−/−^. ^‡^P < 0.05 vs. vehicle treated diabetic ApoE^−/−^, n = 6-8 per group.

### Vascular function

**Figure 2:**
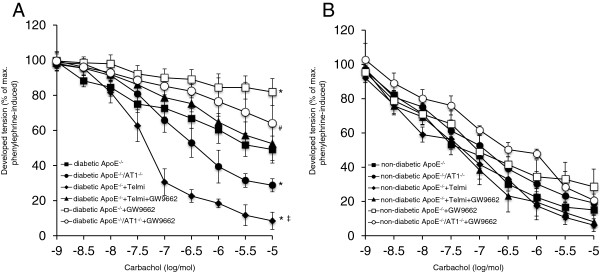
**Vascular function.** After 18 weeks aortic segments of diabetic (**A**) and non-diabetic (**B**) ApoE^−/−^ and ApoE^−/−^/AT1R^−/−^-mice were isolated and their functional performance was assessed in organ chamber experiments. Endothelium-dependent vasodilation induced by carbachol is shown. Diabetic ApoE^−/−^-mice displayed severe impairment of endothelial function compared to ApoE^−/−^/AT1R^−/−^-mice and telmisartan-treated ApoE^−/−^-mice. Treatment of diabetic ApoE^−/−^ and ApoE^−/−^/AT1R^−/−^-mice with GW9662 antagonized the protective vascular effects of AT1R deficiency or AT1 antagonism. ^*^P < 0.05 vs. diabetic ApoE^−/−^, ^#^P < 0.05 vs. diabetic ApoE^−/−^/AT1R^−/−^ and ^‡^P < 0.05 vs diabetic ApoE^−/−^ + Telmisartan + GW9662, n = 6-8 per group.

Vascular function was assessed in isolated aortic ring preparations. In contrast to diabetic ApoE^−/−^/AT1R^−/−^-mice, endothelium dependent vasodilatation was significantly impaired in diabetic ApoE^−/−^-mice indicating that AT1R-deficiency attenuates endothelial dysfunction in diabetic animals. Endothelium-dependent vasodilatation was significantly impaired in GW9662 treated diabetic ApoE^−/−^-mice, whereas treatment with telmisartan led to a significant improvement of endothelium-dependent vasodilatation. Cotreatment with GW9662 abolished the beneficial effect of telmisartan on endothelial function (Figure 2A). In non-diabetic ApoE^−/−^-mice treated with vehicle or telmisartan or telmisartan and GW9662 endothelial function was not significantly affected (Figure 2B). Endothelium independent vasorelaxation induced by nitroglycerin was similar in all groups (data not shown). In addition, vasoconstriction induced by phenylephrine or KCL was similar in all groups (data not shown).

### Vascular oxidative stress

**Figure 3:**
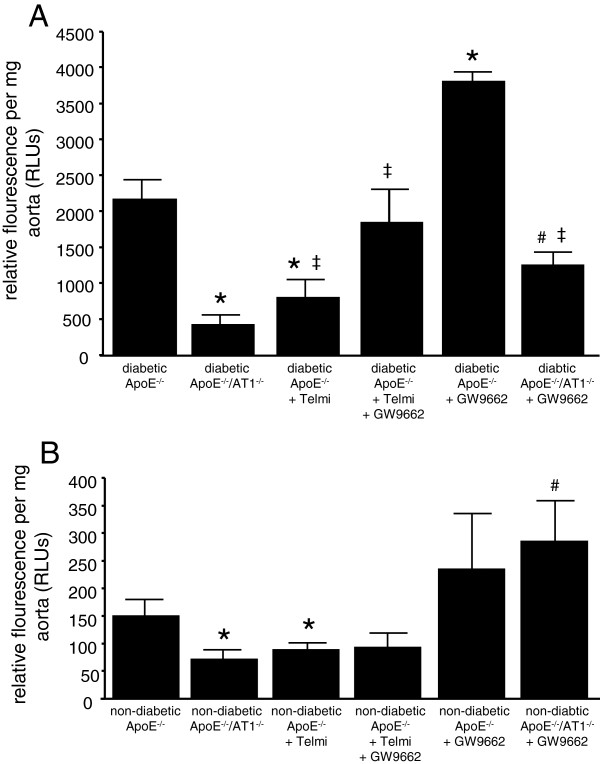
**Oxidative stress.** Vascular ROS formation in isolated aortic segments of diabetic (**A**) and non-diabetic (**B**) ApoE^−/−^ and ApoE^−/−^/AT1R^−/−^-mice were assessed by L-012 chemiluminescence. Diabetic ApoE^−/−^-mice showed enhanced oxidative stress compared to ApoE^−/−^/AT1R^−/−^-mice and telmisartan-treated ApoE^−/−^-mice. Treatment of diabetic ApoE^−/−^ and ApoE^−/−^/AT1R^−/−^-mice with GW9662 raised the levels of oxidative stress to the range found in diabetic ApoE^−/−^-mice. ^*^P < 0.05 vs. diabetic and non-diabetic ApoE^−/−^, ^#^P < 0.05 vs. diabetic and non-diabetic ApoE^−/−^/AT1R^−/−^, ^‡^P < 0.05 vs diabetic ApoE^−/−^ + GW9662, n = 6-8 per group.

Vascular release of ROS radicals was measured by L012-chemiluminescence assays in intact aortic segments. Figure 3A and Figure 3B illustrate that vascular ROS release was significantly higher in diabetic animals than in non-diabetic animals. Diabetic ApoE^−/−^ -mice had significantly higher ROS levels than diabetic ApoE^−/−^/AT1R^−/−^-mice. AT1R-deficiency in diabetic ApoE^−/−^-mice and telmisartan treatment in diabetic ApoE^−/−^-mice significantly decreased vascular ROS release. Co-administration of GW9662 abolished this effect, whereas treatment with GW9662 alone induced the highest ROS release in diabetic ApoE^−/−^-mice (Figure 3A). In non-diabetic animals AT1R-deficiency and treatment with telmisartan reduced vascular ROS release in a comparable fashion (Figure 3B).

### Atherosclerotic lesion formation

**Figure 4:**
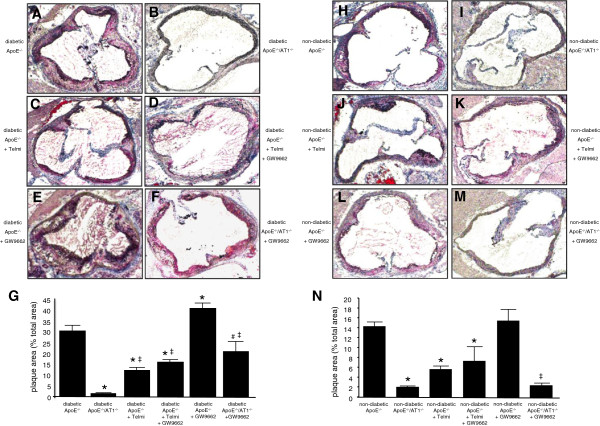
**Atherosclerotic lesion formation.** Diabetic and non-diabetic ApoE^−/−^-mice were treated 18 weeks with telmisartan, GW9662, telmisartan and GW9662 or vehicle, whereas diabetic and non-diabetic ApoE^−/−^/AT1R^−/−^-mice treated with GW9662 or vehicle. Representative histological cross-sections of the aortic root were stained with oil red O to analyse atherosclerotic plaque development, Figure 4A-F (diabetic animals) and Figure 4H-M (non-diabetic animals). Quantitative analysis of atherosclerotic lesion formation indicated as plaque area in % of total area is depicted in Figure 4G (diabetic animals) and Figure 4N (non-diabetic animals). Diabetic ApoE^−/−^-mice displayed increased atherosclerotic lesion formation. AT1R-deficiency and telmisartan treatment in ApoE^−/−^-mice resulted in a significantly reduced area of atherosclerotic lesions, whereas GW9662 antagonized the atheroprotective effects of AT1R deficiency or AT1 antagonism. ^*^P < 0.05 vs. diabetic and non-diabetic ApoE^−/−^, ^#^P < 0.05 vs. diabetic ApoE^−/−^/AT1R^−/−^, ^‡^P < 0.05 vs diabetic and non diabetic ApoE^−/−^ + GW9662, n = 6-8 per group.

Development of atherosclerotic lesions was quantified in diabetic and non-diabetic animals using oil red O-staining and macroscopic analysis of the aortic root after 18 weeks. Figure 4A-F and Figure 4H-M shows representative aortic root preparations of the different groups of animals. In contrast to vehicle treated non-diabetic ApoE^−/−^-mice (Figure 4H), vehicle treated diabetic ApoE^−/−^-mice (Figure 4A) displayed more atherosclerosis in the aortic root. In age-matched diabetic and non-diabetic ApoE^−/−^/AT1R^−/−^-mice, atherosclerotic lesions were significantly diminished (Figure 4B and 4I). Concurrent with the significantly improved endothelial function in telmisartan-treated diabetic ApoE^−/−^-mice (Figure 4C), a significant reduction in atherosclerotic lesion formation was observed compared to vehicle treated diabetic animals. Application of GW9662 in diabetic ApoE^−/−^-mice showed pronounced atherosclerotic lesion formation (Figure 4E). Co-administration of GW9662 and telmisartan attenuated this effect (Figure 4D). In addition, application of GW9662 significantly increased atherosclerotic lesion formation in diabetic ApoE^−/−^/AT1R^−/−^-mice (Figure 4F) compared to vehicle treated diabetic ApoE^−/−^/AT1R^−/−^-mice (Figure 4I). PPARγ inhibition in GW9662 treated diabetic ApoE^−/−^-mice in the highest extent of atherosclerotic lesion formation (Figure 4. In non-diabetic mice (Figure 4H-N) atherosclerotic lesion formation was significantly less pronounced compared to corresponding diabetic groups (Figure 4A-G). AT1R-deficiency lowered atherosclerotic plaque burden in non-diabetic ApoE^−/−^/AT1R^−/−^-mice compared to non-diabetic ApoE^−/−^-mice, indicating that AT1R-deficiency results in decreased atherogenesis not only in diabetic but also in non-diabetic conditions. Subgroup analysis in non-diabetic mice indicated that treatment with telmisartan led to reduced atherosclerotic lesion formation in ApoE^−/−^-mice (Figure 4J), whereas GW9662 (Figure 4L) application showed a tendency towards more atherogenesis. Importantly, as in diabetic animals, AT1R-deficiency mediated significant atheroprotective effects in non-diabetic animals. Vehicle treated ApoE^−/−^/AT1R^−/−^-mice (Figure 4I) had significantly reduced plaque burden compared to ApoE^−/−^ controls (Figure 4H). GW9662 treated ApoE^−/−^/AT1R^−/−^-mice (Figure 4M) had significant less atherosclerotic plaques than ApoE^−/−^-mice treated with GW9662 (Figure 4E). Quantitative analysis of atherosclerotic lesion formation in diabetic and non-diabetic animals is shown in Figure 4G, Figure 4N and in Additional file 1: Table S1.

## Discussion

The present study demonstrates the major role of the AT1R in diabetes-induced atherogenesis. Here we show that AT1R blockade using a pharmacological approach and AT1R deficiency by gene deletion attenuates atherosclerosis and improves endothelial function in diabetic and non-diabetic ApoE^−/−^-mice. In addition, we show for the first time that the antiatherosclerotic effects of AT1R-inhibition are in part mediated via PPARγ *in vivo*. PPARγ-inhibition by GW9662 treatment resulted in enhanced ROS generation and atherosclerotic lesion formation in comparison to untreated diabetic ApoE^−/−^/AT1R^−/−^-mice.

PPARγ belongs to the family of peroxisome proliferator-activated receptors, which control the expression of various genes attributed with potentially vasoprotective effects such as improved glucose metabolism, lipid homeostasis and reduction of oxidative stress [17,18]. Interestingly, PPARγ affects the expression of the AT1 receptor in vascular cells in vitro. Several authors investigated this issue in vascular smooth muscle cells and reported a downregulation of AT1 receptor expression on the transcriptional level following stimulation of PPARγ [14]. Tham et al. analysed the effects of AT1 receptor activation in respect to PPARγ expression in vitro and in vivo and reported a downregulation of PPARγ expression following stimulation with Ang II [19]. This mutual interaction is stressed by clinical observations in patients with metabolic syndrome defined by the coincidence of arterial hypertension and impaired glucose homeostasis [20]. Hypertension and the development of diabetes mellitus might interfere much closer than presently anticipated and the suggested interlocking regulation of PPARy and the AT1 receptor might be the key factor in this respect. On the other hand this interaction might explain why the use of Ang II blockers is associated with improved glucose metabolism. PPARγ activation by PPARγ-agonists induces vascular protection through the improvement of lipid metabolism, anti-inflammation and anti-proliferation [10,18,21]. However, the role of the RAAS in mediating vascular protective effects of PPARγ-agonists is presently not fully understood, particular not in metabolic condition of diabetes [22].

One characteristic of the PPARs is that their activation can occur through a broad spectrum of ligands even with rather low affinity [23]. This implies that particular care must be taken when assessing the PPARγ-dependence of AT1R-signaling-pathway. Signalling pathway connected with PPARγ activation have been investigated in a variety of recent publications [24]. Focussing on vascular inflammation Ji and coworkers [18] found that PPARγ agonist treatment of vascular cells in vitro and in vivo significantly reduced proinflammatory effects of Ang II. The modulatory effects of PPARγ were related to diminished activation of the proinflammatory toll-like receptor 4 (TLR4). TLR4 in turn has been attributed with consecutive activation of the IP10/PKC/NF-*k*B pathway. In contrast to Sugawara et al. PPARγ activation did not affect AT1 receptor expression but significantly reduced AT1 receptor dependent ERK1/2 regulation [14]. Studies published by other authors support the relevant role of the AngII/TLR4-axis in this respect [25].

Our results emphasize that AT1R-PPARγ-interactions are in part responsible for atheroprotective effects of AT1R-deficiency. Application of the specific PPARγ-antagonist GW9662 in diabetic ApoE^−/−^/AT1R^−/−^-mice resulted in enhanced ROS generation and atherogenesis in comparison to untreated diabetic ApoE^−/−^/AT1R^−/−^-mice. However, the here demonstrated ability of GW9662 to inhibit PPARγ does not rule out occurrence of a mechanism independent of PPARγ-inhibition or counteracting AT1R-action by altering its signaling cascade olbeit in the literature, GW9662 has not been associated with pleiotropic pharmacological effects independent of PPARγ-inhibition or associated with AT1R-interference.

Diabetes mellitus is associated with a profound risk of developing atherosclerosis and its complications such as myocardial infarction [26], stroke [27] and peripheral vascular disease [28]. In patients with diabetes, atherosclerotic lesion progression is accelerated if compared to the non-diabetic population [29]. ARBs have recently been shown to prevent the onset of diabetes in hypertensive patients and to reduce cardiovascular and renal disease progression in diabetic patients with hypertension [30-36]. Whether a specific AT1 Blocker shows higher levels of PPARγ agonism and whether this effect results in a clinical benefit remains an unsolved question. Our data showed that telmisartan improved oxidative stress, endothelial function and atherosclerosis while GW9662 treatment showed increased ROS levels with deleterious effects on endothelial function and atherosclerosis. These effects are much more pronounced in diabetic animals compared to non-diabetic animals. This diabetes-dependent aggravation might be partly related to the diabetes-specific over-activation of the RAAS.

In conclusion, AT1R-deficiency or pharmacological inhibition of the AT1R and activation of PPARγ with telmisartan in diabetic individuals have beneficial effects on oxidative stress, endothelial function and atherosclerotic plaque development especially in metabolic and RAAS abnormalities associated with diabetes indicating to a relevant interaction of PPARγ and the RAAS in vivo. These findings suggest a potential utility of AT1R inhibitors with partial PPARγ agonistic activity in the prevention and treatment of diabetic macrovascular complications.

## Competing interests

The authors declare that they have no competing interests.

## Authors' contributions

VT, GN, and SW conception and design of research; VT, AA and DL performed experiments; VT, UMB and CFHM analyzed data; VT, UMB and CFHM interpreted results of experiments; VT and UMB prepared figures; VT, UMB and CFHM drafted manuscript; VT, UMB and CFHM edited and revised manuscript; All authors have read and approved the final version of manuscript.

## Supplementary Material

Additional file 1: Table S1 Absolute values of endothelial funktion, oxidative stress and atherosclerostic plaque development. Click here for file

## References

[B1] Muller WA Diabetes mellitus–long time survival J Insur Med 1998 30 17 27 10186435

[B2] Brownlee M Biochemistry and molecular cell biology of diabetic complications Nature 2001 414 813 820 10.1038/414813a 11742414

[B3] Cooper ME The role of the renin-angiotensin-aldosterone system in diabetes and its vascular complications Am J Hypertens 2004 17 16S 20S 15539106 10.1016/j.amjhyper.2004.08.004

[B4] Candido R Allen TJ Lassila M Cao Z Thallas V Cooper ME Irbesartan but not amlodipine suppresses diabetes-associated atherosclerosis Circulation 2004 109 1536 1542 10.1161/01.CIR.0000124061.78478.94 15023892

[B5] Wassmann S Czech T van Eickels M Fleming I Bohm M Nickenig G Inhibition of diet-induced atherosclerosis and endothelial dysfunction in apolipoprotein E/angiotensin II type 1A receptor double-knockout mice Circulation 2004 110 3062 3067 10.1161/01.CIR.0000137970.47771.AF 15277329

[B6] Gerstein HC Yusuf S Mann JFE Hoogwerf B Zinman B Held C Effects of ramipril on cardiovascular and microvascular outcomes in people with diabetes mellitus: results of the HOPE study and MICRO-HOPE substudy. Heart Outcomes Prevention Evaluation Study Investigators Lancet 2000 355 253 259 10675071

[B7] Kurtz TW Pravenec M Antidiabetic mechanisms of angiotensin-converting enzyme inhibitors and angiotensin II receptor antagonists: beyond the renin-angiotensin system J Hypertens 2004 22 2253 2261 10.1097/00004872-200412000-00003 15614015

[B8] Lindholm LH Ibsen H Borch-Johnsen K Olsen MH Wachtell K Dahlof B Risk of new-onset diabetes in the losartan intervention for endpoint reduction in hypertension study J Hypertens 2002 20 1879 1886 10.1097/00004872-200209000-00035 12195132

[B9] Schupp M Janke J Clasen R Unger T Kintscher U Angiotensin type 1 receptor blockers induce peroxisome proliferator-activated receptor-gamma activity Circulation 2004 109 2054 2057 10.1161/01.CIR.0000127955.36250.65 15117841

[B10] Pang T Benicky J Wang J Orecna M Sanchez-Lemus E Saavedra JM Telmisartan ameliorates lipopolysaccharide-induced innate immune response through peroxisome proliferator-activated receptor-*γ* activation in human monocytes J Hypertens 2012 30 1 87 96 10.1097/HJH.0b013e32834dde5f 22124178 PMC3237779

[B11] Shiota A Shimabukuro M Fukuda D Soeki T Sato H Uematsu E Telmisartan ameliorates insulin sensitivity by activating the AMPK/SIRT1 pathway in skeletal muscle of obese *db*/*db* mice Cardiovasc Diabetol 2012 11 139 10.1186/1475-2840-11-139 23137106 PMC3527353

[B12] Guo Z Zhang R Li J Xu G Effect of telmisartan on the expression of adiponectin receptors and nicotinamide adenine dinucleotide phosphate oxidase in the heart and aorta in type 2 diabetic rats Cardiovasc Diabetol 2012 11 94 10.1186/1475-2840-11-94 22873349 PMC3471013

[B13] Duan SZ Usher MG Mortensen RM Peroxisome proliferator-activated receptor-gamma-mediated effects in the vasculature Circ Res 2008 102 283 294 10.1161/CIRCRESAHA.107.164384 18276926

[B14] Sugawara A Takeuchi K Uruno A Ikeda Y Arima S Kudo M Transcriptional suppression of type 1 angiotensin II receptor gene expression by peroxisome proliferator-activated receptor-gamma in vascular smooth muscle cells Endocrinology 2001 142 3125 3134 10.1210/en.142.7.3125 11416035

[B15] Takeda K Ichiki T Tokunou T Funakoshi Y Iino N Hirano K Peroxisome proliferator-activated receptor gamma activators downregulate angiotensin II type 1 receptor in vascular smooth muscle cells Circulation 2000 102 1834 1839 10.1161/01.CIR.102.15.1834 11023940

[B16] Hsueh W Abel ED Breslow JL Maeda N Davis RC Fisher EA Recipes for Creating Animal Models of Diabetic Cardiovascular Disease Circ Res 2007 100 1415 1427 10.1161/01.RES.0000266449.37396.1f 17525381

[B17] Evans RM Barish GD Wang YX PPARs and the complex journey to obesity Nat Med 2004 10 355 361 10.1038/nm1025 15057233

[B18] Ji Y Lui J Wang Z Liu N Gou W PPARã agonist, rosiglitazone, regulates angiotensin II-induced vascular inflammation through the TLR4-dependent signaling pathway Lab Invest 2009 89 887 902 10.1038/labinvest.2009.45 19451898

[B19] Tham DM Martin-McNulty B Wang YX Wilson DW Vergona R Sullivan ME Angiotensin II is associated with activation of NF-kappaB-mediated genes and downregulation of PPARs Physiol Genomics 2002 11 21 30 12361987 10.1152/physiolgenomics.00062.2002

[B20] Vitale C Mercuro G Castiglioni C Cornoldi A Tulli A Fini M Metabolic effect of telmisartan and losartan in hypertensive patients with metabolic syndrome Cardiovasc Diabetol 2005 15;4 6 15892894 10.1186/1475-2840-4-6 PMC1174877

[B21] Tiyerili V Müller CF Fung S Panek D Nickenig G Becher UM Estrogen improves vascular function via peroxisome-proliferator-activated-receptor-γ J Mol Cell Cardiol 2012 53 2 268 276 10.1016/j.yjmcc.2012.05.008 22634137

[B22] Taguchi I Inoue T Kikuchi M Toyoda S Arikawa T Abe S Node K Pleiotropic effects of ARB on dyslipidemia Curr Vasc Pharmacol 2011 9 2 129 135 10.2174/157016111794519336 21143172

[B23] Desvergne B Wahli W Peroxisome proliferator-activated receptors: nuclear control of metabolism Endocr Rev 1999 20 649 688 10.1210/er.20.5.649 10529898

[B24] Westerink J Visseren F Pharmacological and non-pharmacological interventions to influence adipose tissue function Cardiovasc Diabetol 2011 10 13 10.1186/1475-2840-10-13 21276223 PMC3039566

[B25] Ji Y Liu J Wang Z Lui N Angiotensin II induces inflammatory response partly via Toll-Like receptor 4-dependent signaling pathway in vascular smooth muscle cells Cell Physiol Biochem 2009 23 265 276 10.1159/000218173 19471094

[B26] Beckman JA Creager MA Libby P Diabetes and atherosclerosis: epidemiology, pathophysiology, and management JAMA 2002 287 2570 2581 10.1001/jama.287.19.2570 12020339

[B27] Kissela BM Khoury J Kleindorfer D Woo D Schneider A Alwell K Epidemiology of ischemic stroke in patients with diabetes: the greater Cincinnati/Northern Kentucky Stroke Study Diabetes Care 2005 28 355 359 10.2337/diacare.28.2.355 15677792

[B28] Fadini GP Miorin M Facco M Bonamico S Baesso I Grego F Circulating endothelial progenitor cells are reduced in peripheral vascular complications of type 2 diabetes mellitus J Am Coll Cardiol 2005 45 1449 1457 10.1016/j.jacc.2004.11.067 15862417

[B29] Renard C Van Obberghen E Role of diabetes in atherosclerotic pathogenesis. What have we learned from animal models? Diabetes Metab 2006 32 15 29 10.1016/S1262-3636(07)70243-4 16523183

[B30] Andraws R Brown DL Effect of inhibition of the renin-angiotensin system on development of type 2 diabetes mellitus (meta-analysis of randomized trials) Am J Cardiol 2007 99 1006 1012 10.1016/j.amjcard.2006.10.068 17398202

[B31] Geng DF Jin DM Wu W Xu Y Wang JF Angiotensin receptor blockers for prevention of new-onset type 2 diabetes: A meta-analysis of 59,862 patients Int J Cardiol Int J Cardiol 2012 155 2 236 242 10.1016/j.ijcard.2010.10.011 21036409

[B32] Julius S Kjeldsen SE Weber M Brunner HR Ekman S Hansson L Outcomes in hypertensive patients at high cardiovascular risk treated with regimens based on valsartan or amlodipine: the VALUE randomised trial Lancet 2004 363 9426 2022 2031 10.1016/S0140-6736(04)16451-9 15207952

[B33] Yusuf S Pfeffer MA Swedberg K Granger CB Held P McMurray JJV Effects of candesartan in patients with chronic heart failure and preserved left-ventricular ejection fraction: the CHARM-Preserved Trial Lancet 2003 9386 777 781 13678871 10.1016/S0140-6736(03)14285-7

[B34] Watanabe M Inukai K Sumita T Ikebukuro K Ito D Kurihara S Effects of telmisartan on insulin resistance in Japanese type 2 diabetic patients Intern Med 2010 49 17 1843 1847 10.2169/internalmedicine.49.3189 20823643

[B35] Sakamoto M Suzuki S Hayashi T Iuchi H Isaka T Sakamoto N Effects of candesartan in hypertensive patients with type 2 diabetes mellitus on inflammatory parameters and their relationship to pulse pressure Cardiovasc Diabetol 2012 11 118 10.1186/1475-2840-11-118 23034088 PMC3489584

[B36] Nishida Y Takahashi Y Nakayama T Asai S Comparative effect of angiotensin II type I receptor blockers and calcium channel blockers on laboratory parameters in hypertensive patients with type 2 diabetes Cardiovasc Diabetol 2012 11 53 10.1186/1475-2840-11-53 22594344 PMC3416676

