# Supplementary material for: AT1-receptor-deficiency induced atheroprotection in diabetic mice is partially mediated via PPARγ
Source: Cardiovasc Diabetol. 2013 Feb 1;12:30. doi: 10.1186/1475-2840-12-30 (PMC3667017; doi:10.1186/1475-2840-12-30)
Supplement: Additional file 1: Table S1 — Absolute values of endothelial funktion, oxidative stress and atherosclerostic plaque development. [file 1475-2840-12-30-S1.docx]

| **Supplement Table 1** | Endothelial function (max. Relaxation %) | Oxidative stress (RLUs/per mg aorta) | Atherosclerotic plaque development (%) |
| --- | --- | --- | --- |
| **Diabetic ApoE^-/-^**  Vehicle  Telmisartan  Telmisartan+GW9662  GW9662 | 49±8  8±05  52±16  82±9 | 2153±282  804±243  1847±461  3809±121 | 30±4  12±2  15±2  40±4 |
| **Diabetic ApoE/AT1^-/-^**  Vehicle  GW9662  **Non-diabetic ApoE^-/-^**  Vehicle  Telmisartan  Telmisartan+GW9662  GW9662  **Non-diabetic ApoE/AT1^-/-^**  Vehicle  GW9662 | 29±4  64±9  5±6  6±4  8±3  20±11  19±5  20±7 | 423±125  1253±176  151±28  90±13  94±24  236±88  72±16  286±75 | 4±1  21±5  14±1  6±1  7±3  15±3  2±1  2±1 |

**Supplement Table 1:**

Absolute values of endothelial funktion, oxidative stress and atherosclerostic plaque development.
